# High-Performance Liquid Chromatography (HPLC) Method Validation for Identifying and Quantifying Rebamipide in Ethosomes

**DOI:** 10.7759/cureus.56061

**Published:** 2024-03-12

**Authors:** Dina Kako, Mowafaq M Ghareeb, Mohammed S Al-Lami

**Affiliations:** 1 Pharmaceutics, University of Duhok, Duhok, IRQ; 2 Pharmaceutics, University of Baghdad, Baghdad, IRQ; 3 Pharmaceutics, University of Basra, Basrah, IRQ

**Keywords:** kinetics of drug release, dd solver, lipid-based nanovesicles, ich, hplc, validation, ethosomes, rebamipide

## Abstract

Introduction

The research aimed to develop a robust, high-performance liquid chromatography (HPLC) analytical method for the quantitative assessment of rebamipide encapsulated in ethosomes. Rebamipide, a quinolinone derivative, holds promise as a therapeutic agent for dry eye, but challenges such as low bioavailability and vision clouding post-installation have prompted innovative approaches. Encapsulation in ethosomes, lipid-based nanovesicles, offers a potential solution to enhance ocular bioavailability.

Materials and methods

The study focused on creating a specific, linear, accurate, precise, and robust HPLC method, addressing entrapment efficiency (%EE), drug content, and drug release of rebamipide in prepared ethosomes. Statistical validation followed International Conference of Harmonization (ICH) specifications. The method's parameters were evaluated within a concentration range of 4-24 µg/ml, with recovery rates indicating accuracy and low % relative standard deviation (RSD) values confirming precision. Limits of detection (LOD) and quantification (LOQ) for rebamipide were determined.

Results

After preparing the ethosome dosage form by film hydrating method for rebamipide, the rebamipide entrapment efficiency in ethosomes was established at 76% ± 7, while the drug content was found to be 93% ± 6. The drug release process demonstrated zero-order kinetics and five different models of kinetics were applied for a comprehensive analysis. The method exhibited excellent system suitability, specificity, and linearity. Recovery rates for rebamipide ranged from 90% to 100%, and repeatability was confirmed by low %RSD values. The LOD and LOQ for rebamipide were determined to be 1.04 μg/mL and 3.16 μg/mL, respectively.

Conclusion

The developed HPLC method proved suitable for the quantitative determination of rebamipide in ethosomes, offering rapid and accurate analysis. The results underscore the method's specificity, accuracy, and precision within the specified concentration range. Overall, the validated method contributes to the advancement of ocular drug delivery systems, providing a reliable analytical tool for pharmaceutical research.

## Introduction

Rebamipide is a novel quinolinone derivative that Otsuka Pharmaceutical Company (Tokyo, Japan) developed and synthesized [1]. After being discovered, it was applied as a substance that aided in the lesions healing in a rat research model of stomach ulcers experiment. Mucosta® tablet (Otsuka Pharmaceutical Company, Tokyo, Japan) was the brand name under which it was sold for the treatment of stomach ulcers starting in 1990 [2].

Following the discovery that rebamipide's pharmacological properties included enhancing gastric mucus (mucin), research conducted on the impact of the medication on ocular surface mucin prompted its subsequent utilization in the creation of a therapeutic intervention for dry eye syndrome. In addition, rebamipide improved corneal and conjunctival mucin as well as conjunctival goblet cells in rabbits, according to nonclinical investigations [3]. The logP and pKa values of rebamipide were reported as 2.9 and 3.3, respectively. Additionally, rebamipide has been classified as a class IV medication according to the Biopharmaceutical Classification System (BCS). Based on these results, Japan introduced rebamipide for dry eye treatment in January 2012 (Mucosta ophthalmic suspension unit dose 2%) [4,5]. Dry eye is a chronic multifactorial disease of the tears and ocular surfaces [6]. Patients in the clinical trial had rebamipide instillations four times daily, and clinical tests have shown that rebamipide is beneficial in reducing the symptoms and indicators of keratoconjunctivitis sicca [7,8]. Topical drops are widely recognized as the prevailing method for giving ocular drugs due to their practicality, safety, instant efficacy, patient compliance, non-invasiveness, and comfort, but only a small fraction, specifically 20% or 7 μL, of the administered dose remains in the precorneal region following topical application, due to the limitations of the eyes such as anatomic and physiological (reflex blinking) causing most of the topically applied dose to be lost consequently resulting on low bioavailability in order to get a satisfactory therapeutic outcome, it is necessary to administer frequent instillations [9,10]. Furthermore, after receiving the milky white fluid with a 2% rebamipide concentration, the patient will have clouded vision right away [11]. In order to decrease patient compliance and increase ocular bioavailability, rebamipide can be encapsulated in a nanostructured system (ethosomes), which is the vesicular system that has received the most extensive investigation. These have greater deformability, softness, and elasticity since they are lipid-based nanovesicles. Ethosomes are multilamellar nanovesicles composed of ethanol and phospholipids [12]. When developing this kind of formulation, it is critical to control the entrapment efficiency (%EE) and drug release [13]. Therefore, a method for analytically determining the drug content, %EE, and drug release determination by five different kinetics models of drug release (zero order, first order, Higuchi model, Korsmeyer-Peppas model, and Hixson-Crowell model) within 12 hours is essential. There are currently few published analytical methods for determining rebamipide [14,15]. The objective of this investigation is to present a validated method for estimating and measuring drug entrapment efficiency, drug content, and drug release in ethosomes using a quick, sensitive, and accurate HPLC analytical methodology. The statistical validity of the devised method was confirmed in accordance with the international conference of harmonization International Conference of Harmonization (ICH) requirements [16]. The HPLC method was evaluated with respect to their linearity, accuracy, precision, specificity, limit of detection (LOD), limit of quantitation (LOQ), and robustness.

## Materials and methods

Instrumentation

An Agilent Technologies 1260 Infinity HPLC with a 1260 quat pumpVL (Agilent Technologies, Santa Clara, California) and for chromatographic separation, an ultraviolet-visible (UV-vis) detector was utilized. A 1260ALS injector loop (Agilent Technologies) and a 50 µL syringe were used as part of a manual injector system. OpenLab software (Agilent Technologies) managed the HPLC equipment and data collection, and a kromaphase C18 (250 -X 4.6 mm; 10 µm particle size) column was employed. pH meter, analytical balance (Hanna Instruments, Smithfield, Rhode Island), Chromafil pore size 0.45µm, filter membrane 25mm, and nylon syringe filter 0.22 mm (Macherey-Nagel, Düren, Germany) were used for sample preparations. Digital ultrasonic (bath sonicator; iSonic, Chicago, Illinois) and a hot air oven (Binder Inc., Bohemia, New York) were used for ethosome preparation. A Hermle Z 383 K centrifuge (Hermle AG, Gosheim, Germany) was also used for drug content determination. A dialysis bag cut-off (8000-12000 MWT) and a hotplate (Cole-Parmer, Vernon Hills, Illinois) for in vitro drug release determination were used.

Materials

Analytical-grade rebamipide and soy lecithin were procured from Apollo Healthcare Resources (Singapore). The purity of analytical-grade rebamipide typically exceeds 98%, while soy lecithin of analytical or research grade often achieves a purity level of 95%. Di-sodium hydrogen phosphate and sodium dihydrogen phosphate anhydrous, utilized in the investigation, are generally available in analytical grades with purities around 98%. HPLC-grade acetonitrile and ethanol are commonly obtained with purities of 99.9%.

Greenness study

This greenness study underscores our commitment to conducting environmentally conscious research, emphasizing sustainable practices at every stage. From the selection of ingredients and adoption of pollution-free processes to the use of eco-responsible glassware and non-hazardous chemicals, our approach prioritizes the preservation of the environment. The choice of biyrex glassware, known for its durability and reusability, aligns with our goal of reducing waste and promoting a circular economy. By meticulously considering the ecological impact of our work, we strive to set a standard for responsible scientific inquiry that minimizes its carbon footprint and fosters a harmonious relationship with the environment.

Preparation of buffer

About 11.9gm of sodium dihydrogen phosphate anhydrous and 14gm of disodium hydrogen phosphate were dissolved and completed to 1L of distilled water separately, then the pH was adjusted to 6.2 by mixing 407ml and 92.5ml from the above-prepared solutions consequently, and the volume was completed to 1L by distilled water. The solution undertook filtration using a Chromafil pore size of 0.45µm and a 25mm filter membrane. Additionally, it was subjected to ultrasonic degassing for a duration of 15 minutes.

Methods

Chromatographical Conditions

The method employed and developed in this study was isocratic elution. A mixture was prepared by combining 300 mL of phosphate buffer solution with a pH of 6.2 and 750 mL of water. A total of 170 mL of acetonitrile of HPLC-grade was added to 830 mL of the prepared solution. This resulting solution was then utilized as the mobile phase. This specific composition of the mobile phase, with the inclusion of phosphate buffer, plays a crucial role in promoting the effective separation of rebamipide. The phosphate buffer contributes to maintaining the desired pH, facilitating ionization, and enhancing the chromatographic behavior of the compound. Furthermore, the addition of HPLC-grade acetonitrile aids in achieving a balance between solubility and chromatographic resolution for rebamipide, flowing at a rate of 1 mL/min through a KromaPhase C18 column (Scharlab Internacional, Barcelona, Spain) with sizes of 250 mm in dimension and 4.6 mm in width, containing particles with a size of 10 µm. The hydrophobic nature of the C18 column, combined with the properties of the mobile phase, ensures excellent interaction with the analyte, resulting in accurate and reliable HPLC analysis of the rebamipide drug. For each run, a sample size of 20 µL was injected and analyzed using a UV detector set at a wavelength of 222 nm while maintaining a temperature of 35°C.

Calibration Curve and Standard Solution Preparation

The concentration of 0.4 mg/mL was achieved by adding 40 mg of rebamipide to a 100 mL volumetric flask, dissolving it, and subsequently diluting it with phosphate buffer pH 6.2 until the desired concentration was reached. Phosphate buffer pH 6.2 was used to dilute the standard solution to yield 4, 8, 12, 16, 20, and 24 µg/mL rebamipide concentrations [17]. The concentration to rebamipide area ratio was used to fit a calibration curve.

Validation of the analysis approach

The System Suitability Test

By injecting six repetitions of the standard solution concentration of 12 µg/ml, a system suitability test was carried out to make it clear we chose this concentration to ensure accurate reading by the HPLC, after working on the calibration curve, we deal with the same concentration. Peak area and retention period had a relative standard deviation % relative standard deviation (RSD), which was one of the observed metrics. The %RSD for peak area and retention period must be less than 2% for the test to succeed [18].

Specificity

In order to ascertain that the excipients included in the formulations did not cause any interference, an evaluation of the specificity of the HPLC method was conducted. The specificity was considered by injecting the excipients and comparing them with the drug chromatogram. The absence of a peak that appears close to or during the medication retention period is the acceptable criterion [19]. Investigations were conducted into the potential for drug and excipient peak interference.

Linearity

A test is said to be linear if it produces results that are inversely relative to the concentration of the analyte being tested for. Three injections at each of six distinct concentrations of rebamipide (4, 8, 12, 16, 20, and 24 µg/ml) were used to determine linearity. Peak areas, on average, were graphed vs concentrations. The calibration curve was then used to compute the coefficient of correlation, slope, and intercept, which were used to assess linearity. As a rule of thumb, if the rate of the correlation coefficient (r2) is greater than 0.998, the data fit to the regression line has been considered to be satisfactory [20].

Accuracy

The degree to which the observed value is similar to the predicted value expresses how accurate an analytical process is. The percentage of analyte recovered (R%) is used to determine this value. Successive analysis (n=6) was completed for three different concentrations (4 µg/ml, 12 µg/ml, and 20 µg/ml) of standard rebamipide solution to assess the reliability of the established method. The experiment's results were statistically evaluated using the created formula to examine the recovery and applicability of the developed method. Acceptable average recovery lies between 90% and 110% [10]. 

Recovery% = (recovered concentration /injected concentration) × 100

Precision

The concept being referred to is the degree of concordance observed among several measurements conducted on identical homogenous materials within controlled conditions. The achievement of this outcome can be facilitated by conducting an evaluation of the method's repeatability and intermediate precision. The study examined the repeatability, also known as intraday precision, by analyzing standard rebamipide solutions with low (4 μg/mL), medium (12 μg/mL), and high (20 μg/mL) concentrations. This analysis was conducted on the same day, with six replicates for each concentration. The investigation of intermediate precision, also known as interday precision, involved assessing the three concentration levels of standard rebamipide solutions in sextuplicate on three separate days. The recorded data were expressed as the relative standard deviation, which should be less than 2% [21,22].

The limit of detection (LOD) and the limit of quantification (LOQ)

The limit of detection (LOD) is the smallest detectable quantity of an analyte in a sample, which does not always require measurement. Alternatively, the lowest amount of analyte that can be accurately and precisely quantified is known as the limit of quantification (LOQ). The LOD and LOQ of the method were determined by utilizing the slope (S) of the calibration curve and the lowest standard deviation (SD) obtained from the response, as per the equations. The aforementioned action was carried out in compliance with the rules set forth by the International Council for Harmonisation of Technical Requirements for Pharmaceuticals for Human Use (ICH) [23]. LOD = 3.3 × SD/S; LOQ = 10 × SD/S, where calibration curve slope = S and response standard deviation (peak area) = SD.

Robustness

As recommended in the ICH guidelines, a measure of an analytical procedure's dependability under typical settings is its robustness or its resistance to being impacted by small but deliberate changes in method limitations. To assess the robustness of the analytical method, a systematic evaluation of critical parameters was undertaken, necessitating adjustments to the initially employed isocratic elution method. The modifications included transitioning from a phosphate buffer solution with a pH of 6.2 to pH 7, incorporating an acetonitrile: phosphate buffer ratio of 1.7:8.3, adjusting the flow rate to 1.5 mL/min, and maintaining the column temperature at 25°C. These variations in mobile phase pH, flow rate, and column temperature were systematically introduced to gauge their impact on the chromatographic performance. This robustness study provides a comprehensive understanding of the method's tolerance to fluctuations in operational conditions, ensuring its reliability and suitability for routine analytical applications. Percent recovery and %RSD values were obtained [24].

Ethosome preparation

Ribamipide-loaded ethosomes have been prepared by a modified film hydration method [25], where the 80mg of soy lecithin phosphatidylcholine, ribamipide (40 mg), as a lipid phase, and ethanol solution (20 % v/v) as hydration phase were used; to put it simply, the lipid mixture was dissolved in 10mL of ethanol, which was then left in the hot air oven at 50 degrees Celsius for 24 hours. This resulted in a thin coating of dry lipids on the container. In order to reconstitute the film, 10mL of 20% aqueous ethanol was added with stirring, and a sonicator was used for vesicle formation and size reduction for 10 minutes.

Entrapment efficiency (%EE) and drug content determination

The %EE is the proportion of rebamipide encapsulated within vesicles in the ethosome formulations. The determination of rebamipide %EE in ethosomes formulation was conducted using an indirect method [26]. The samples (n=6) were subjected to centrifugation for a duration of 60 minutes at a rotational speed of 4000 rotations per minute (rpm) at a temperature of 4°C using a centrifugal filter device. The filters were extracted and afterward diluted with a 100 mL volume of phosphate buffer at a pH of 6.2. From this solution, 3 mL was further diluted with an additional 100 mL of phosphate buffer at the same pH. The amount of unentrapped medication was determined by utilizing the following equation:

%EE = ((total amount of the drug-the amount of the drug in the supernatant)/(total amount of the drug)) × 100

Furthermore, the concentration of rebamipide in ethosomes was determined by preparing dilutions with phosphate buffer pH 6.2 (n=6) and subsequently analyzing them for the total amount of drug present in the formulations. The amount of rebamipide entrapped in the ethosomes and the total drug content in the formulations were estimated using HPLC/UV-visible spectroscopy with a detector set at 222 nm, following a standard calibration curve.

In vitro drug release

The dialysis bag approach [27] was used to investigate the in vitro release of rebamipide from the manufactured ophthalmic ethosomes. The drug equivalent of 40 mg was weighed and then inserted in a sealed dialysis bag with a molecular weight cut-off between 8000 and 12000 MWT. The dialysis bag was placed in a glass beaker containing 800 mL of phosphate buffer of pH 6.2, and the beaker was kept at 37°C under 200 rpm over a hotplate. At regular intervals (during the course of 12 hours), 5 mL samples were obtained from the beaker and immediately replaced to keep the sink condition constant. The rebamipide concentration in the samples was calculated using spectrophotometry at 220 nm. There were three separate runs of the study. The obtained results for the in vitro release data were fitted into multiple mathematical models, including the zero order, first order, Higuchi model, Korsmeyer-Peppas model, and Hixson-Crowell model [28] to explore the kinetics and mechanism of drug release of rebamipide from the generated ethosomes formulations by applying DDSolver software [29] to the data.

## Results

System suitability test

The system suitability test conducted in this HPLC method analysis is essential for evaluating and ensuring the reliability of the chromatographic system. Parameters such as retention time and retention area were closely monitored, meeting predetermined acceptance criteria. Deviations from expected values could signal potential issues, prompting corrective actions. The system suitability test results not only validate the method but also guide ongoing analyses, ensuring the system's capacity for accurate and precise data generation over time. The values derived from the measurements were found to satisfy the acceptance criteria. The retention time and retention area exhibit a relative standard deviation (RSD) of less than 2%, as presented in Table 1.

**Table 1:** The outcome of the suitability examination of 12µg/ml rebamipide using HPLC systems HPLC - high-performance liquid chromatography; AUC - area under the curve; SD - standard deviation; RSD - relative standard deviation

RSD%	Average ± SD (n=6)	Parameters
0.42	16.7 ± 0.07	Retention time (minutes)
0.6	814.6 ± 5.6	Area under curve (AUC)

Specificity

**Figure 1:**
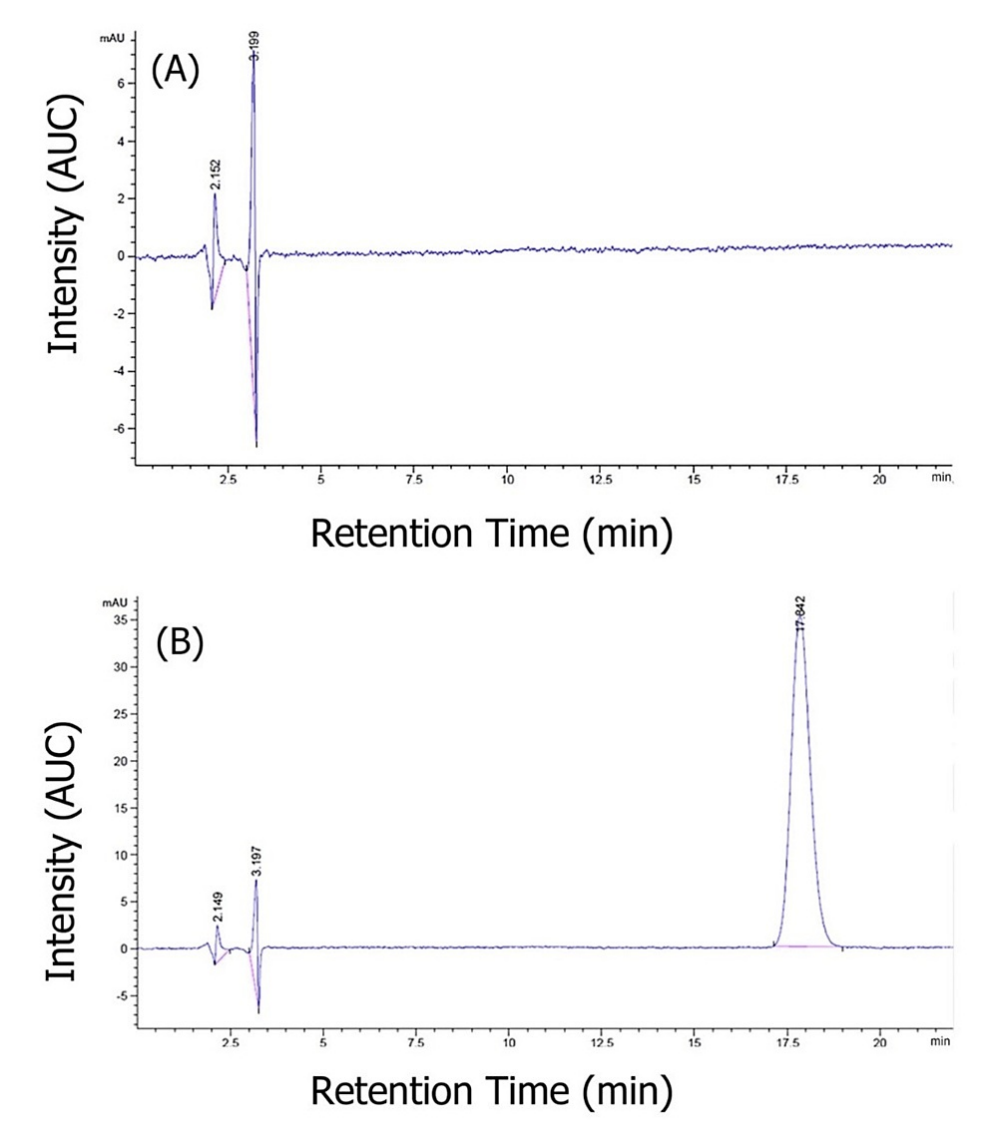
A) chromatogram of ethosome matrix; B) chromatogram of rebamipide ethosomes AUC - area under the curve

The method's specificity has been examined by studying the ethosome matrix and ethosomes of rebamipide. A peak in close proximity to the retention time of rebamipide was not recognized from 16.5 to 17.6 min at 222 nm, which verified that the approach has a significant level of specificity (Figure 1).

Linearity

**Figure 2:**
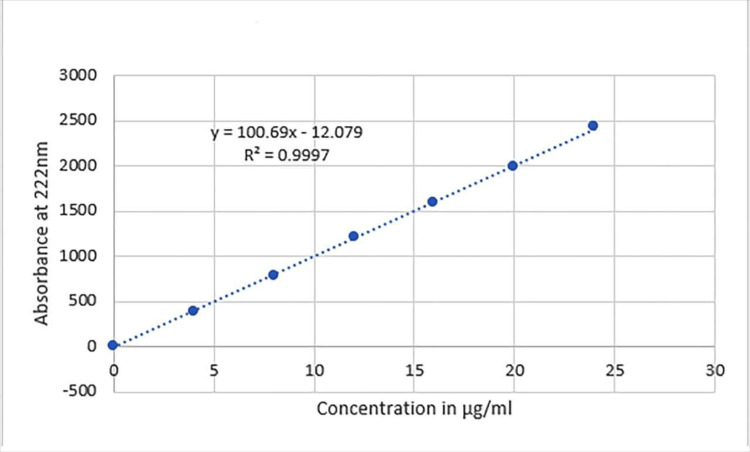
Standard curve of rebamipide

A strong positive correlation (r >0.999) was found between the area under the curve (AUC) of rebamipide and the corresponding values ranging from 4-24 μg/mL (Figure 2). The obtained linear regression equation is y=100.69x-12.079. The correlation coefficient of 0.9997 suggests a strong linear relationship between the variables, showing a high degree of linearity.

Accuracy

Recovery experiments determined the method's accuracy. The % recovery and % relative standard deviation were computed following three recovery studies (n=6) from the collected data (Table 2). The resulting percent recovery value satisfies the criteria for being between 90 and 110, and %RSD of less than 2%. The HPLC analysis method exhibited high accuracy, as evidenced by the consistent alignment of measured values with true values for the analytes. These findings affirm the suitability of the HPLC method for accurate quantitative analysis, which is essential for its credibility in diverse applications.

**Table 2:** Recovery measurements of rebamipide SD - standard deviation; RSD - relative standard deviation

%RSD	% recovery	Concentration measured µg/ml ± SD (n=6)	Concentration in theory µg/ml
0.61	90%	3.6 ± 0.02	4
0.87	98%	11.59 ± 0.11	12
0.67	99%	19.71 ± 0.14	20

Precision

Replicate analyses consistently demonstrated excellent precision, as indicated by minimal variability and low relative standard deviations (RSDs) across multiple measurements of the same sample. The requisite precision (%RSD <2) was achieved in both intra- and inter-day repeatability and intermediate precision tests (Table 3). The demonstrated precision underscores the dependability of the HPLC method, ensuring the consistency and reliability of the quantitative data obtained throughout the study.

**Table 3:** Rebamipide intra- and inter-day precision study SD - standard deviation; RSD - relative standard deviation

%RSD	Concentration measured µg/ml ± SD (n=6)	Actual concentration µg/ml	Daytime
0.05	3.1 ± 0.04	4	1^st^ day
0.02	11.5 ± 0.19	12	
0.01	20.0 ± 0.15	20	
0.04	3.7 ± 0.07	4	2^nd^ day
0.01	11.8 ± 0.11	12	
0.00	20.0 ± 0.07	20	
0.02	3.5 ± 0.04	4	3^rd^ day
0.00	11.8 ± 0.05	12	
0.01	19.9 ± 0.12	20	

The limit of detection (LOD) and the limit of quantification (LOQ)

The LOQ signal-to-noise ratio was 10, whereas the LOD signal-to-noise ratio was 3. Rebamipide's 1.04 µg/mL LOD and 3.16 µg/mL LOQ were determined. The determination of the limit of detection (LOD) and limit of quantification (LOQ) is crucial for assessing the sensitivity and reliability of the analytical method employed in this study. The LOD, defined as the lowest analyte concentration that can be reliably detected but not necessarily quantified, was established through rigorous statistical analysis. Our findings reveal an LOD value that underscores the method's ability to discern trace amounts of the analyte with a high degree of confidence.

Similarly, the limit of quantification (LOQ), representing the lowest concentration at which the analyte can be accurately and precisely quantified, was determined using established protocols. The obtained LOQ value reflects the method's capability to provide precise quantitative measurements within the specified concentration range. The low values of both LOD and LOQ signify the method's sensitivity and efficacy in detecting and quantifying the analyte, even at minimal concentrations.

It is imperative to note that these values were ascertained under strict experimental conditions, ensuring the method's reliability and reproducibility. The establishment of LOD and LOQ provides valuable insights into the analytical method's performance, guiding researchers and practitioners in the interpretation of results and ensuring the method's suitability for applications demanding high sensitivity and accuracy.

Robustness

The study assessed the robustness of an analytical methodology to determine its resilience to minor parameter variations (Table 4). Results showed that slight deviations from specified conditions did not significantly impact results. This robustness demonstrates the method's reliability, adaptability, and reproducibility in diverse laboratory settings. The outcomes showed that the RSD value is <1, demonstrating that the robustness criteria have been met.

**Table 4:** Rebamipide robustness test with a concentration of 12µg/mL in terms of RSD value (n=6) RSD - relative standard deviation

Initial conditions	Variation conditions	Measured concentration µg/mL	Recovery%	RSD%
Mobile phase: acetonitrile pH 6.2: phosphate buffer ratio of 1.7:8.3	Mobile phase: acetonitrile pH 7: phosphate buffer ratio of 1.7:8.3	11.64	97	0.3
Flow rate: 1ml/min	Flow rate: 1.5ml/min			
Temp.: 35 c^o^	Temp.: 25 c^o^			

Entrapment efficiency %EE and drug content

The percentage of rebamipide that is contained in vesicles and included in the ethosome formulations is known as the EE percent, and similarly, the total amount of drugs in formulations are present in Table 5, which shows the obtained result of %EE and drug content which was measured by the developed method of analysis according to ICH. The validated HPLC method proved highly effective for the quantification of rebamipide in ethosomal formulations, filling a notable void in available analytical techniques. As no existing HPLC method was found for the specific quantification of rebamipide in such formulations, the developed method demonstrated its significance and novelty in addressing this analytical gap. Its successful application in the quantification of rebamipide within ethosomal matrices not only establishes a valuable contribution to the field but also opens avenues for further exploration and utilization of the validated method in pharmaceutical research and formulation development.

**Table 5:** Entrapment efficiency and drug content of rebamipide (n=6) SD - standard deviation; EE - entrapment efficiency

Formula	%EE ± SD	Drug content ± SD
Rebamipide ethosomes (n=6)	76 ± 7	93 ± 6

In vitro drug release study

**Figure 3:**
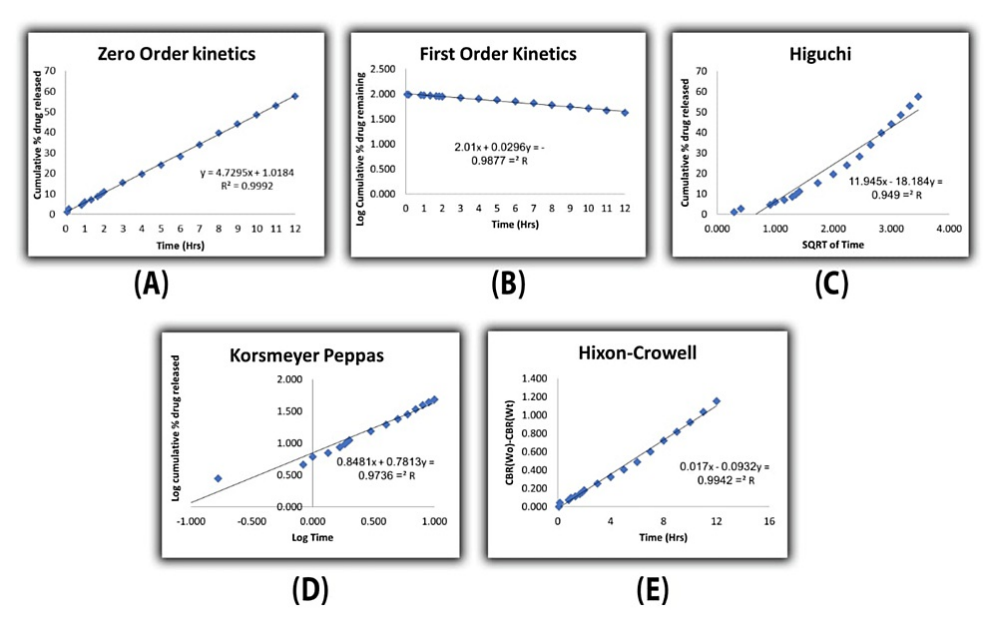
In vitro rebamipide release A) zero order, B) first order, C) Higuchi model, D) Korsmeyer-Peppas model, and E) Hixson-Crowell model

Upon fitting the in vitro drug release data to various kinetic models, namely zero order, first order, Higuchi, Korsmeyer-Peppas, and Hixon-Crowell, as depicted in Figure 3, it was observed that the formulation under investigation predominantly adhered to zero order kinetics. This conclusion was drawn from the discernment that the zero-order kinetic model exhibited the highest correlation coefficient (R) among the tested models. This finding suggests that the release of rebamipide from the formulation occurs independently of the cumulative drug concentration, indicating a consistent and controlled release pattern over time. The rigorous analysis of kinetic profiles enhances our understanding of the release mechanisms, facilitating precise formulation optimization and therapeutic efficacy prediction in pharmaceutical applications.

## Discussion

Based on our understanding from previously published articles about nanoparticles, we created a lipid-based nanocarrier system for rebamipide because it is crucial for enhancing the bioavailability of poorly soluble drugs, achieving targeted and controlled release, and minimizing side effects. Our research sheds light on the ethosome, while existing studies have focused on liposomes to reach the same purpose [30,31].

Validating a new, simple, and reliable method for determining the %EE and drug release of rebamipide was even more significant due to the absence of a specific HPLC method for the detection of rebamipide in specific dosage forms. This method revealed an EE of developed ethosome at approximately 76% ± 7, with the drug content measured at 93% ± 6. The drug release process followed zero-order kinetics, meaning the drug is released at a constant rate regardless of the initial concentration. This was analyzed using five different kinetic models. Our findings are corroborated by the works of Mishra et al. and Venugopal et al., who similarly demonstrated zero-order drug release from ethosomes [32,33]. This will result in decreased patient compliance with the suspension dose form of rebamipide according to the ICH Q2(R2) guidelines on the validation of analytical procedures [23].

Our findings for the HPLC method were evaluated with respect to their linearity, accuracy, precision, specificity, LOD, LOQ, and robustness within acceptable ranges and this study [10,34], which added further support to our findings. The %RSD of both retention time and area was found to be less than 2%. Furthermore, no interference peak was seen at the retention time of rebamipide. The proposed analytical approach was determined to be appropriate and selective for the quantification of the medication in ethosomes. The approach exhibited a high degree of linearity within the concentration range of 4-24µg/mL; by recovery studies, the validity of the planned method was assessed, and the results demonstrated outstanding recoveries of 90-110% for rebamipide, suggesting that the procedure is accurate; additionally, the repeatability of the newly created method was demonstrated by a significantly low value of (%RSD less than 2%), hence affirming the method's adequate precision under identical operating conditions. The resolution of the chromatogram for the system suitability solution was acceptable under all of the deliberately changed chromatographic settings.

An RSD of less than 2% with the absence of significant alterations in the chromatographic parameters serves as evidence for the robustness of the recently formulated approach. The method developed for quality control analysis of any drug has been demonstrated to possess sensitivity, as indicated by the LOD and LOQ values of 1.04 μg/mL and 3.16 μg/mL, respectively, for rebamipide. The robustness of the newly devised approach is demonstrated by the observation of a relative standard deviation of less than 2% and the absence of any substantial alterations in the chromatographic parameters. The results of this study demonstrate that the HPLC methodology is capable of effectively detecting and quantifying rebamipide inside ethosome nanoparticles.

## Conclusions

In summary, the establishment and validation of an HPLC method for the quantitative analysis of rebamipide in ethosomes have been successfully accomplished. The method demonstrated excellent specificity, linearity, accuracy, precision, and robustness, meeting the requirements set forth by ICH guidelines. With its simplicity and efficiency, this analytical approach offers a valuable means of assessing rebamipide content in nanocarriers. Additionally, the determination of entrapment efficiency and drug release can be conveniently and accurately performed using this method. Overall, this HPLC method serves as a reliable and practical tool for the reliable analysis of rebamipide in ethosomes, contributing to the advancement of pharmaceutical research and development. Based on the developed method, we recommend further analysis of the formulated ethosome for its sensitivity, in vitro and in vivo penetration to ocular surfaces, and effectiveness in managing dry eye diseases. This is crucial to ensure that the formulated ethosome can improve patient compliance or we can increase patient adherence compared to conventional dosage forms by lowering application frequency.
